# Microbial Aerosols in Livestock Farming Environment: A Threat That Cannot Be Ignored

**DOI:** 10.3390/vetsci12121147

**Published:** 2025-12-01

**Authors:** Hao Lu, Yuqing Xie, Longxin Chen, Yue Song, Limeng Zhang, Runting Li, Xiaoning Nie, Yichen Liu, Guoqiang Zhu, Xueyan Ding, Linqing Wang

**Affiliations:** 1 Molecular Biology Laboratory, Zhengzhou Normal University, Zhengzhou 450044, China; 2 College of Veterinary Medicine, Henan Agricultural University, Zhengzhou 450046, China; 3 College of Veterinary Medicine, Yangzhou University, Yangzhou 225009, China; 4 Department of Life Science, Zhengzhou Normal University, Zhengzhou 450044, China

**Keywords:** livestock breeding environment, microbial aerosols, sources, hazards, influencing factors, prevention and control measures

## Abstract

Under modern intensive and large-scale animal farming practices, the high animal density and confined spaces have progressively elevated the risk of disease transmission while complicating epidemic prevention and control. Current estimates indicate that over 70% of animal disease risks stem from viral infections, primarily transmitted via aerosol pathways. Such aerosol-mediated transmission not only triggers concentrated disease outbreaks within farms but also endangers farm workers and adjacent environments, thereby amplifying prevention challenges and substantially impeding the sustainable development of livestock industries. Therefore, this review systematically expounds the sources, hazards, and influencing factors of microbial aerosols in livestock farming environments. Furthermore, it proposes targeted prevention and control measures to address these challenges, aiming to provide theoretical and practical insights for enhancing disease prevention and promoting the healthy development of the livestock industry.

## Simple Summary

Intensive livestock production systems face mounting biosecurity threats from aerosol-transmitted pathogens. These airborne transmission mechanisms create cascading risks—compromising animal welfare, occupational safety, and environmental health—which collectively undermine industry sustainability. Microbial aerosols originate from external environments, livestock (respiratory emissions, rumen microbes), feces, and related activities (bedding, vaccines). The key factors influencing aerosol transmission include particle size, meteorological conditions, transmission distance, animal activity, and housing environments. These aerosols cause animal disease outbreaks and economic losses and threaten human health via zoonotic pathogens. Prevention and control measures involve source control (optimal density, waste management), disinfection (UV, chemicals, ozone), and balanced ventilation/insulation. This review aids in risk management for sustainable livestock farming.

## 1. Introduction

The farming environment plays a pivotal role in the sustainable development of the livestock industry and the quality of its products. A well-managed environment not only ensures animal health and optimal productivity but also safeguards the well-being of surrounding communities and ecosystems. However, intensive farming practices, characterized by high population densities and confined spaces, lead to elevated concentrations of diverse pollutants, including organic and inorganic dust, harmful gases, and microbial aerosols [1]. These pollutants pose significant risks to animal health and hinder the progress of the livestock industry.

Microbial aerosols are relatively stable dispersion systems formed by microorganisms adhering to solid or liquid particles suspended in a gaseous medium. The diameter of these particles typically ranges from 0.001 to 100 μm [2], with their size directly influencing the health of the respiratory systems in humans and animals. Particles larger than 10 μm are primarily deposited in the nasal cavity, those between 5–10 μm reach the lower respiratory tract, particles smaller than 5 μm can penetrate the lungs, and those below 2 μm can enter the bronchioles and alveoli [3,4]. Microbial aerosols can be classified based on their composition into viral, bacterial, and fungal aerosols. Among these, viral aerosols are a significant mode of transmission for many viruses in livestock farming environments, characterized by rapid spread, long-distance dissemination, and challenges in control [5].

Microbial aerosols are highly transmissible in intensive livestock farming environments, often leading to concentrated outbreaks of infectious diseases among animals [6]. These outbreaks result in significant animal mortality and substantial economic losses for the livestock industry [7]. Furthermore, zoonotic viruses transmitted via aerosols can spread over large areas, contaminating the environment and posing serious health risks to farm workers and nearby communities. Notable examples include the global SARS outbreak in 2003, which affected 26 countries; the 2009 H1N1 influenza pandemic [8]; and the 2013 H7N9 avian influenza outbreak, which rapidly spread across 42 cities in China [9]. These events underscore the critical role of microbial aerosols in the transmission of zoonotic diseases. This review focuses on the sources, hazards, influencing factors, and mitigation strategies related to microbial aerosols in intensive livestock farming environments, aiming to provide insights for both theoretical research and practical disease control efforts.

## 2. Sources of Microbial Aerosols in Livestock Farming Environments

**Figure 1:**
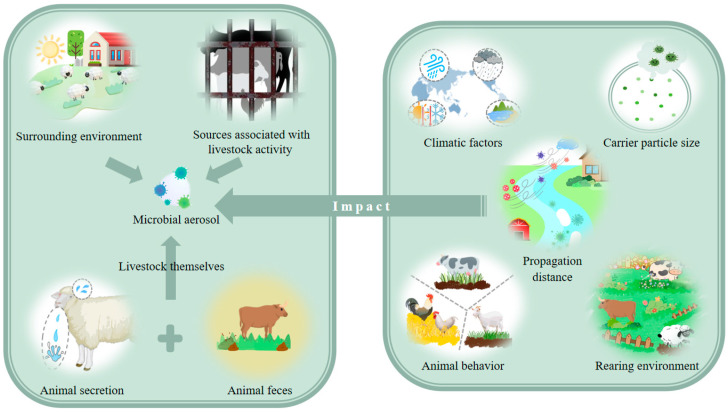
The sources of microbial aerosols and the factors influencing their transmission. The left part presents the sources of microbial aerosols, including the surrounding environment, sources associated with livestock activity, animal secretion, and animal feces. The right part illustrates the factors influencing their transmission, covering climatic factors, carrier particle size, propagation distance, animal behavior, and rearing environment. This figure was created by BioGDP.com [10].

Microbial aerosols in livestock farming environments originate from multiple sources, including external environmental introduction, pathogen emissions from infected animals, microbial release during fecal decomposition, and activity-related factors such as bedding materials, vaccine usage, and animal secretions (Figure 1).

### 2.1. External Sources

The external environment of animal houses can be a potential source of microbial aerosols. The extent to which these aerosols infiltrate the indoor environment is dependent upon factors such as aerosol particle size, wind conditions, and the design and efficiency of the ventilation system. Natural wind currents can transport microbial aerosols over considerable distances, while mechanical ventilation systems may draw outdoor aerosols into indoor environments, particularly under favorable wind direction and speed conditions. Once inside, microbial aerosols can further disperse through internal air circulation, increasing the risk of cross-contamination within different areas of the housing facility [11,12,13].

After floating in the air for a certain period, microbial aerosols will settle onto various surfaces both inside and outside livestock houses due to gravity, such as floors, walls, and equipment surfaces. When livestock, poultry, or breeders come into contact with these surfaces, microorganisms may enter the bodies of livestock via contact transmission or be carried to other areas [14]. Microorganisms can also adhere to transportation devices, breeders’ bodies, work clothes, etc., and spread through these carriers [15]. For instance, when biosecurity measures (e.g., mandatory clothing changes and disinfection) are not strictly implemented, pathogens can enter livestock buildings via aerosols and be further disseminated within the farm on the clothing of personnel. When they enter the house to work, the microorganisms may be transmitted to livestock, poultry, or the indoor environment through contact [16]. Meanwhile, transport vehicles and tools may carry microorganisms when entering and leaving the farm, serving as a medium for the spread of microorganisms between different farms [11,17]. Improper design or poor management of the air intake and exhaust ports, doors, and windows of livestock houses can also become channels for microbial spread. For instance, if the exhaust port is not filtered or disinfected, microbial aerosols may be directly discharged into the external environment. If the air intake lacks effective filtration, external microbial aerosols may directly enter the house, increasing the risk of microbial spread [18,19].

### 2.2. Livestock Themselves

The presence of diseases, especially viral diseases, in livestock themselves is a crucial factor contributing to the increase in the concentration of microbial aerosols in the indoor environment. The rumen of ruminants is a complex ecological system rich in microorganisms, which play an important role in the digestion process. However, when the animal is infected with a virus, the microbial community in the rumen may serve as a host for the virus [20,21]. The virus can replicate and proliferate through the interaction between the microorganisms and the rumen wall. When the animal belches, that is, expels gas during the rumination process, these virus-carrying microorganisms have the opportunity to be expelled from the body along with the gas and enter the air in the livestock house, thus forming a potential source of aerosol transmission [22,23].

Apart from belching, sick livestock can also release a large number of viruses through respiratory behaviors such as coughing and sneezing. Research indicates that a single cough or sneeze can generate up to 1 × 10^5^ bio-droplets [24]. These droplets contain viruses, which can remain suspended in the air and disperse with the air currents, increasing the risk of virus transmission within the shed and infection of other animals. The spread of viral aerosols depends not only on the amount of virus emissions but is also influenced by factors such as the ventilation conditions, humidity, temperature, and animal density within the shed [25,26,27,28,29].

Livestock manure is not only a source of organic matter in the farm environment but also a crucial generator of microbial aerosols [30]. Animal feces contain a rich microbial community, including bacteria, viruses, fungi, and parasites. During the decomposition of feces, these microorganisms can be released into the air to form aerosols, thereby affecting the biosecurity of both the indoor environment of the livestock house and the surrounding areas [30].

Studies have revealed a high degree of similarity between the microbial communities in the air of pig housing and those in pig feces, indicating that fecal microorganisms can enter the air through pathways such as excretion, desiccation, and dust dispersion, forming microbial aerosols [31,32]. In calf housing, the accumulation of feces leads to a significant increase in airborne anaerobic bacteria, which are closely associated with gastrointestinal health [33]. This rise not only reflects the direct impact of feces on air quality but may also signal underlying intestinal health issues in calves. Similarly, microbial contamination in poultry housing is a significant concern, with research showing that culturable bacteria from chicken feces can reach concentrations as high as 1 × 10^3^ cfu/m^3^ in the air. This highlights feces as a major source of microbial pollution and underscores its potential impact on air quality [34]. Alarmingly, studies have identified a 100% similarity between *Escherichia coli* strains isolated from chicken feces and those in the air, demonstrating the potential for fecal pathogens to spread via aerosols, posing risks to both animal and human health.

### 2.3. Sources Associated with Livestock Activity

In the livestock farming environment, the generation of bio-aerosols is not limited to feces and respiratory secretions. It also encompasses substances such as animal urine, shed hair, sweat, sebaceous gland secretions, feed, bedding materials, etc. [35]. These collectively form a complex and multi-sourced bio-aerosol generation system. In particular, bedding materials mixed with feces and urine act like natural culture media, providing an abundant growth environment for microorganisms such as fungi and bacteria, thus becoming a hotbed for microbial attachment and growth [36]. Microorganisms in the bedding materials multiply rapidly under favorable conditions, forming dense biological populations. When the bedding materials dry out or are affected by wind, these microorganisms, along with tiny particles in the bedding, are carried into the air, forming dust or aerosols, which then spread to the external environment and become one of the important sources of indoor bio-aerosols [37,38]. Although shed hair and sebaceous gland secretions may not directly carry pathogens, under specific conditions, such as in a high-humidity and high-temperature environment, they can also create favorable conditions for microbial growth, indirectly facilitating the formation of bio-aerosols [39,40].

Moreover, in the livestock production process, the use of vaccines, especially when immunization is carried out via routes such as nasal drops, aerosolization, and eye-dropping, can lead to a situation where the live microorganisms in the vaccines, after a certain period in the animal’s body, may be excreted through the respiratory tract or skin, forming pathogenic microbial aerosols [41]. This phenomenon is particularly pronounced when live vaccines are used. Although live vaccines can provide an immune effect that more closely mimics natural infection, the potential risk of aerosol transmission associated with them cannot be overlooked. Immunized animals may become carriers of pathogenic microbial aerosols for a certain period, increasing the risk of transmission among animals within the same group or across species [42,43,44].

## 3. Hazards of Microbial Aerosols in Livestock Farming Environments

**Figure 2:**
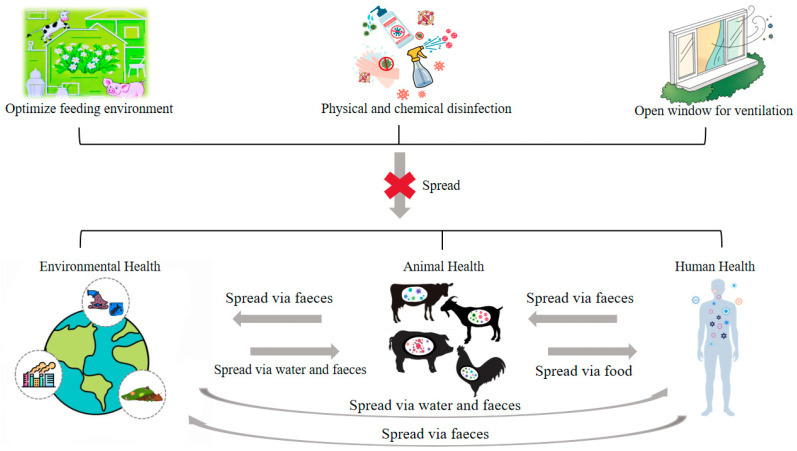
The hazards and prevention-control measures of microbial aerosols. The prevention and control measures in the upper part include optimizing the feeding environment, physical and chemical disinfection, and opening windows for ventilation, which can effectively block the spread of microbial aerosols. The lower part presents the hazard pathways of mutual transmission of microbial aerosols among environmental, animal health, and human health through pathways such as feces, water, and food. This figure was created by BioGDP.com [10].

Livestock-derived microbial aerosols pose dual threats: they trigger acute epidemics (e.g., avian influenza, Newcastle disease) and chronic immunosuppression in animals through glucocorticoid-mediated pathways [45,46,47], while simultaneously elevating respiratory disease risks for workers (chronic obstructive pulmonary disease, pneumonia) and enabling cross-regional pathogen dispersion (>10× ambient bacterial/fungal loads) that jeopardizes surrounding communities [48,49,50] (Figure 2).

### 3.1. Adverse Effects on Animal Health

Microbial aerosols carry a variety of pathogenic microorganisms such as *Staphylococci*, aerobic spore-forming bacteria, *Streptococci*, and *Enterobacteria*. These can form a stable suspension system in the air and easily breach the natural defenses of organisms. This is particularly severe for poultry [51,52]. Avian influenza virus (AIV) can be efficiently transmitted through microbial aerosols, spreading rapidly among poultry flocks and triggering large-scale epidemics [53]. This not only threatens the lives of poultry but also poses a significant risk to public health security [54]. Besides avian influenza, diseases such as Newcastle disease, infectious laryngotracheitis, and bronchitis also use microbial aerosols as vectors, accelerating their spread in enclosed spaces and causing severe economic losses and social panic [19].

Furthermore, livestock that are exposed to high-concentration non-pathogenic microbial aerosols over an extended period, although not immediately stricken by life-threatening diseases, will gradually slip into a sub-healthy state. They exhibit symptoms such as listlessness, reduced appetite, a significant decline in production performance, and an increased culling rate. In this condition, abnormal secretions will appear in the sensitive parts of the livestock, such as the eyes, mouth, and nose. This not only affects their appearance but also impairs the animals’ physiological functions and welfare levels [50,55]. Even at lower concentrations, the persistent presence of microbial aerosols can trigger complex immune response mechanisms in poultry. Among these, the most crucial impact is on the neuro-endocrine system. It stimulates the body to release a large amount of adrenocortical (glucocorticoid) hormones, which are typical products of the stress response [56]. However, an excessive amount of these hormones instead induces an immunosuppressive effect, leading to a decline in the natural immunity of livestock, weakening their ability to combat external pathogens. At the same time, this also disrupts the normal growth and metabolism processes, further slowing down the growth rate of livestock. Moreover, high-concentration adrenocortical (glucocorticoid) hormones can also reduce the effectiveness of vaccines, weaken the post-vaccination immune response, and lower the originally expected protective effect [19].

### 3.2. Impact on the Breeding Staff and the Surrounding Environment

Microbial aerosols in livestock housing pose significant health risks not only to the animals housed but also to the workers managing these facilities. For farm employees frequently exposed to environments rich in microbial aerosols, every breath carries potential hazards. This occupational group faces a substantially higher burden of respiratory diseases compared to the general population [57], with chronic obstructive pulmonary disease, lung infections (such as pneumonia), bronchitis, and various respiratory inflammations being particularly prevalent [58]. The underlying mechanism involves the continuous deposition of airborne microbial particles in the lungs through inhalation, leading to cumulative tissue damage and functional decline. Over time, this results in markedly reduced respiratory efficiency and diminished quality of life [45,59].

Within the enclosed livestock barns, the concentration of microbial aerosols far exceeds what one might imagine. The content of bacteria and fungi is particularly astonishing, being several times or even dozens of times that of the external environment [60]. These minute yet densely packed biological particles not only form a thick suspension within the barn but, propelled by the wind, also cross the enclosures and spread to a broader area, posing a non-negligible threat to the ecological environment surrounding the farm and the health of nearby residents [61,62].

In the face of the environmental pollution and health risks posed by microbial aerosols, it is of utmost importance to adopt effective monitoring and prevention-control measures [63]. Livestock farms should conduct regular monitoring of microbial aerosols to understand the trends in their concentration changes and promptly implement measures to reduce emissions. Meanwhile, the ventilation and purification systems in livestock barns should be enhanced. By leveraging high-efficiency filtration technologies, the generation and spread of microbial aerosols can be mitigated [19]. For the surrounding residential areas, it is advisable to increase the greenbelt. Harnessing the natural purification function of plants, a green protective barrier can be established [64,65].

## 4. Factors Influencing the Transmission of Microbial Aerosols in Livestock Farming Environments

The concentration, viability, and transmission dynamics of microbial aerosols in livestock farming environments are multifactorial, critically influenced by carrier particle size, meteorological conditions, propagation distance, animal-specific factors, and husbandry practices (Figure 1).

### 4.1. The Size of Carrier Particles

The transmission of microbial aerosols in livestock farming environments is closely linked to the size of their carrier particles, a phenomenon that underscores the physical basis of microbial dispersal in the air. Carrier particles, including dust, fecal matter, respiratory droplets, hair, and bedding fragments, not only provide a substrate for microbial attachment but also influence the suspension and dispersal dynamics of these microorganisms. As the size of carrier particles increases, so does the viral load and survival capacity they can support, thereby enhancing the persistence and range of microbial aerosol transmission [66,67].

In a study on air sample collection from isolation rooms of infected pigs, Alonso et al. found that the influenza A virus (IAV), porcine reproductive and respiratory syndrome virus (PRRSV), and porcine epidemic diarrhea virus (PEDV) released by the infected pigs could disperse among various particles with particle sizes ranging from 0.4 to 10 μm [68]. Notably, the virus concentration reached its peak in particles with a size of 9–10 μm. The concentrations of IAV, PRRSV, and PEDV reached 4.3 × 10^5^, 5.1 × 10^4^, and 3.5 × 10^8^ copies/m^3^, respectively [68]. Within infected poultry houses, the virus becomes airborne by adsorbing to carrier particles consisting of dried feces, feather debris, and feed dust. Larger particles (>5 μm) exhibit high settling velocities, leading to localized environmental contamination and serving as the primary driver of high-concentration, short-range transmission [69]. Conversely, smaller particles (<2.5 μm) can form persistent aerosols that are transported over long distances by poultry house exhaust and wind. Both modeling and field evidence confirm that these fine aerosols containing HPAIV can travel several kilometers downwind to infect susceptible flocks and initiate new outbreaks [70,71]. This finding provides critical insights into viral transmission within livestock environments. For instance, in poorly ventilated poultry or swine barns, large dust particles originating from feed, feces, and dander can act as stable carriers for pathogens such as IAV, HPAIV and PRRSV. These particles facilitate the airborne spread of viruses within and between facilities, thereby elevating the risk of cross-contamination and disease outbreaks [38].

### 4.2. Meteorological Factor

The transmission of microbial aerosols is also associated with meteorological factors. Relative temperature, relative humidity (RH), ultraviolet (UV) radiation, and wind can influence the settling time of particles, thereby affecting the trajectory of microbial aerosols [68]. Among these factors, temperature and humidity have interactive effects on biological aerosols, with their combined influence causing inactivation of proteins and membrane phospholipids [12]. Hermann et al. demonstrated that PRRSV aerosols are more stable under low-temperature or low-RH conditions, and temperature has a greater impact on the half-life of PRRSV than RH [72]. Schuit et al. investigated the effects of simulated sunlight on the survival of the influenza virus in biological aerosols under RH conditions of 20% and 70%, respectively [73]. They found no significant differences between the two RH levels, while sunlight exposure was negatively correlated with the transmission of influenza aerosols. Ijaz et al. reported that the half-life of the coronavirus HCoV-229E in aerosols (defined as the time required for a 50% reduction in infectivity) was approximately 3 h at 20 °C ± 1 °C and RH of 80% ± 5% [74]. In contrast, the virus exhibited longer half-lives in intermediate humidity conditions (68 h at RH = 50% ± 5% and 26 h at RH = 30% ± 5%). Additionally, the half-life of HCoV-229E at low temperatures (6 °C ± 1 °C) was significantly longer than that at room temperature, indicating that low temperatures favor the survival of HCoV-229E under equivalent humidity conditions. Moreover, air movement also affects the transmission of aerosols [75].

### 4.3. The Length of the Propagation Distance

The transmission of microbial aerosols is inherently tied to their dispersal distance, with viruses released by infected animals capable of widespread distribution in the air within livestock housing, surrounding farm areas, and live animal markets. Confined livestock facilities often exhibit high concentrations of viral aerosols, which further disperse through airflow, contaminating ambient environments. A previous study by Brito et al. detected PRRSV at concentrations of 10^6^ TCID_50_/mL in air samples 30 m downwind of a U.S. swine farm [76], while Alonso et al. identified PEDV at levels up to 10^5^ copies/m^3^ within 0.02–16.09 km downwind of multiple U.S. swine operations [77]. Overall, regions with high farm and animal densities may exhibit elevated viral detection rates, concentrations, and diversity in ambient air. However, aerosol-borne viruses gradually lose concentration and infectious potential over distance. For instance, Li et al. and Corzo et al. reported that IAV concentrations in outdoor air near swine barns were 1–3 orders of magnitude lower than indoor levels [8,78]. As IAV traveled 0.9–2.1 km downwind from exhaust fans, its concentration decreased by one order of magnitude, and the proportion of infectious IAV in positive samples dropped from 1.6% to 0%. These findings highlight the critical role of dispersal distance in modulating aerosol-mediated disease risks.

### 4.4. The Activities of the Animals Themselves

The generation and transmission of biological aerosols in livestock farming are influenced by a combination of factors, including the animals’ activity level, species, age, health status, and inherent disease susceptibility, among others. These factors not only affect the concentration of biological aerosols but also determine their composition and structure, thereby impacting indoor environmental quality [11,79,80,81].

The activity level of animals is positively correlated with the concentration of biological aerosols. In livestock farming environments, daytime is the period of most intense animal activity. During this time, behaviors such as respiration, movement, and feeding are significantly increased, leading to a substantial rise in the generation and release of biological aerosols within the barns. For example, during foraging, walking, and wallowing, pigs and chickens can disturb dust, bedding, and feces in the barn, causing microbes, viruses, and particulate matter to become suspended in the air and form biological aerosols. Additionally, the respiratory process of animals also generates a large amount of respiratory secretions, which further increases the concentration of biological aerosols [82,83].

The differences in livestock species significantly influence the particle size, concentration, and microbial community structure of biological aerosols within animal housing [18]. Variations in the physiological structure, husbandry practices, and metabolic by-products among different livestock species collectively determine the characteristics of biological aerosols. For instance, biological aerosols in swine barn houses exhibit distinct microbial compositions. Swine barn aerosols may contain higher levels of *Escherichia coli* and *Salmonella* spp., whereas poultry house aerosols may be enriched with avian influenza virus and avian leukemia virus [60,84]. Additionally, the differences in fecal and urinary composition among livestock species also affect the generation and transmission of biological aerosols [12,19,60,85].

The age of livestock also exerts a significant influence on the concentration and composition of biological aerosols within animal housing. Research has shown that as livestock age increases, changes in metabolic activity and physiological functions lead to a gradual rise in the concentration of biological aerosols [86,87]. For example, the immune systems of young livestock are not yet fully developed, resulting in relatively lower concentrations of viruses and bacteria in their feces and respiratory secretions [88,89]. However, with increasing age, enhanced metabolic activity in livestock leads to higher microbial content in excreta. Moreover, increased contact frequency among livestock raises the risk of viral and bacterial transmission, thereby contributing to the elevated concentration of biological aerosols within the barns [89,90].

Livestock health status and inherent disease susceptibility are key factors regulating biological aerosols in animal housing. Healthy animals maintain intact mucosal barriers and balanced gut microbiota, which limit the shedding of pathogenic microorganisms [91]. In contrast, those with acute or chronic diseases such as PRRS and avian influenza, as well as subclinically infected individuals, release significantly more pathogens through respiratory secretions and feces. Symptomatic PRRS-infected pigs, for instance, shed 10–100 times higher viral loads in aerosols [92]. Inherent disease susceptibility shaped by breed and genetics further modulates aerosol dynamics: commercial broilers are more prone to avian pathogenic Escherichia coli than native chicken breeds [93,94], while swine with *TLR4* or *CD163* gene polymorphisms show increased susceptibility to PRRSV, both leading to enhanced pathogen shedding [95,96]. Genetically resistant breeds, on the other hand, shed fewer microorganisms, reducing the pathogenicity and concentration of biological aerosols and collectively influencing disease transmission.

### 4.5. Management of Feeding Environment

The biological aerosols in livestock farming environments are influenced by a variety of husbandry conditions, which determine not only their concentrations but also their composition and transmission patterns, thereby having significant impacts on animal health and indoor environmental quality.

The use and type of bedding materials are among the key factors influencing biological aerosols within livestock housing. Bedding serves as the primary environment for animal activities, and its physical and chemical properties, such as hygroscopicity and porosity, provide a conducive environment for microbial growth. Different types of bedding materials, such as straw, wood shavings, and sand, exhibit significant differences in microbial load and composition, thereby affecting the composition of biological aerosols within the barns. For example, fresh and dry bedding can reduce microbial growth and lower the concentration of biological aerosols, whereas damp and aged bedding can become a breeding ground for pathogenic microorganisms, increasing the generation of biological aerosols [33,97,98]. Therefore, implementing strict litter management is a critical biosecurity measure. This includes the regular removal and replacement of damp or soiled bedding, coupled with the prompt disposal of carcasses to eliminate decomposition-related contaminants. Furthermore, these practices should be incorporated into a systematic cleaning and disinfection protocol to comprehensively reduce the load of aerosolized microorganisms.

The mode of animal husbandry also significantly affects the concentration and composition of biological aerosols within livestock housing. Cage rearing and floor rearing (free-range) are two common husbandry methods. Under cage-rearing conditions, the microbial diversity of biological aerosols in poultry houses is often higher than that in free-range conditions. This is because cage rearing reduces the contact between chickens and the ground, thereby decreasing the direct emission of microorganisms from feces and bedding. However, the dense arrangement of chickens and the accumulation of feces can increase the concentration of ammonia and microorganisms in the air, forming a specific composition of biological aerosols [87]. Therefore, targeted biosecurity measures are indispensable. In cage housing systems, this necessitates regular removal of manure beneath the cages and enhanced ventilation to reduce airborne pollutants. Furthermore, strict carcass disposal protocols and routine disinfection of cages and equipment are crucial to prevent pathogen accumulation, collectively forming a comprehensive aerosol management strategy.

Proper ventilation can effectively reduce the concentration of biological aerosols within animal housing and improve air quality. Ventilation not only affects the morphology and concentration of most particles in biological aerosols but also determines their spatial distribution within the housing [79]. For example, effective longitudinal ventilation can reduce the residence time of biological aerosols, thereby decreasing microbial accumulation. In contrast, poor ventilation conditions may lead to excessively high concentrations of biological aerosols in localized areas within the housing, increasing the risk of disease transmission among livestock [79].

Manure management and rigorous disinfection are pivotal in regulating bioaerosol dynamics. The effective and frequent removal of waste directly eliminates a primary source of microbial amplification, functioning as a source control measure that limits bioaerosol production. In parallel, routine disinfection, utilizing approaches like UV irradiation and chemical agents, serves as a residual mitigation measure by inactivating persistent microorganisms in the environment. This combined approach significantly reduces the overall bioaerosol load, thereby protecting animal health and forming an essential defense against airborne microbial threats [1,99].

## 5. Potential Mitigation Strategies of Microbial Aerosols in Livestock Farming Environments

**Figure 3:**
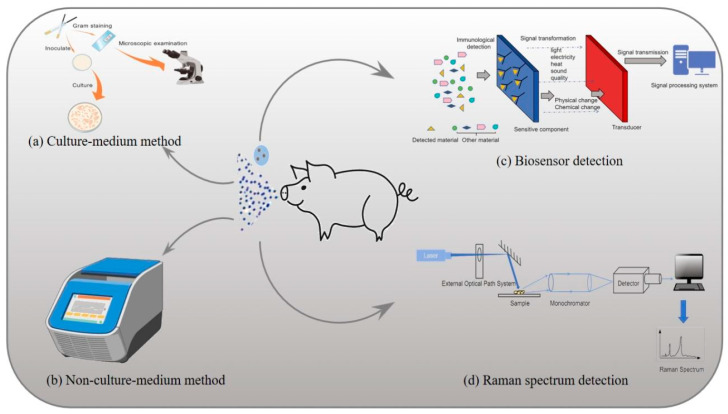
Schematic diagram of microbial aerosol detection technology. (**a**) Bacterial species identification is achieved through the examination of colony morphology, staining characteristics, growth features and biochemical reactions of the bacteria. (**b**) PCR technology is utilized for molecular biological identification based on bacterial nucleic acids. (**c**) Specific biological molecules, such as antibodies, nucleic acid probes, or enzymes, are employed to recognize and bind target microorganisms. The presence of microorganisms is then quantitatively or qualitatively analyzed by detecting changes in signals. (**d**) Based on the Raman scattering effect, in the detection of microbial aerosols, Raman spectroscopy can be used to identify specific molecular vibrations of microorganisms, thereby enabling the identification and quantitative analysis of microbial species. This figure was created by BioGDP.com [10].

Preventive strategies against microbial aerosols in livestock farming environments encompass source control, transmission blocking, and balanced thermal-ventilation management, prioritized through context-specific implementation to ensure optimal efficacy (Figure 2). Moreover, implementing corresponding control measures only after detecting microbial aerosols in livestock environments (common detection techniques are illustrated in Figure 3) and evaluating their distribution and concentration may yield superior containment efficacy.

### 5.1. Prevention and Control at Source

Controlling the generation of microorganisms at the source within livestock housing is a crucial step in ensuring animal health and improving breeding efficiency. The core of this strategy lies in optimizing the breeding environment to reduce the proliferation of pathogenic microorganisms, thereby lowering the risk of animal infections. The following are specific measures for source control, aimed at providing livestock farmers with a comprehensive management guide.

Firstly, rational stocking density is an essential prerequisite for controlling the proliferation of microorganisms. Overcrowding can increase contact between animals, thereby facilitating the spread of pathogens. Therefore, stocking density should be scientifically determined based on the species, growth stage, and physiological needs of livestock, ensuring that each animal has optimal living space to reduce stress reactions and immune system compromise caused by overcrowding [100]. Secondly, timely removal of feces, litter, and bedding materials is crucial for minimizing microbial growth. Feces and urine from livestock serve as breeding grounds for microorganisms. Regular cleaning and prompt replacement of bedding can effectively reduce the accumulation of pathogens. It is recommended to use methods such as dry manure removal or water-soaked manure, combined with regular bedding replacement, to ensure the cleanliness and hygiene of the housing environment. Additionally, adopting new breeding technologies such as microbial fermentation beds, which utilize the decomposition action of beneficial microorganisms, can further reduce the growth of harmful microorganisms while improving indoor air quality [64]. Lastly, regular disinfection is indispensable [101]. Choosing appropriate disinfectants and applying them at the prescribed concentrations and methods to disinfect the interior and exterior of livestock housing, breeding tools, and staff can significantly reduce the microbial load and decrease the incidence of diseases. Disinfection should be coordinated with the breeding cycle, such as conducting thorough disinfection after the removal of livestock, to create a safe environment for the next breeding cycle [64].

### 5.2. Transmission Route Control

Disinfection, as an essential means of interrupting the transmission pathways of microorganisms, plays an irreplaceable role in controlling disease spread and safeguarding public health. Different types of microorganisms, such as viruses and bacteria, exhibit varying degrees of resistance to disinfectants, necessitating the adoption of appropriate disinfection strategies tailored to different scenarios and targets. For instance, African Swine Fever Virus (ASFV) is a highly resilient pathogen with significant economic impact. Its exceptional environmental persistence is a key epidemiological feature; the virus can remain infectious for extended periods in various matrices, including contaminated feed, feces, and on surfaces such as steel and rubber for days to weeks under certain conditions [102,103]. These features pose new challenges and demands for disinfection efforts [104,105]. Disinfection technologies can generally be categorized into physical and chemical methods, each with specific applications and advantages.

#### 5.2.1. Physical Disinfection

Physical disinfection technologies, including UV irradiation, electrostatic precipitation, ionizing radiation, laser exposure, and microwave radiation, leverage physical agents to directly disrupt microbial structural integrity or critical physiological functions, thereby achieving microbial inactivation.

##### UV Radiation

UV irradiation is one of the most widely used methods for air disinfection. When microorganisms such as bacteria and viruses are exposed to UV light, their DNA or RNA absorbs photon energy, leading to the formation of thymine dimers, which inhibit the replication capability of the microorganisms, cause genetic material loss, and result in either vegetative cell death or reproductive cell death [106]. Moreover, UV light with a wavelength of 254 nm has the best sterilization effect [107]. In livestock farming, this technology is primarily applied within ventilation systems. By installing UV lamps in exhaust or air intake ducts, it can inactivate airborne pathogens before contaminated air is recirculated or released into the environment, thereby helping to control the spread of infectious aerosols indoors. In a study focusing on aerosolized PRRSV—a primary transmission route for the virus—the U.S. Department of Agriculture (USDA) confirmed that conventional 254 nm UV-C requires a dose of merely 0.0872 mJ/cm^2^ to achieve 99% inactivation of airborne PRRSV [108]. In comparison, 222 nm far-UV-C exhibits even higher efficiency, achieving the same 99% inactivation with just 0.0429 mJ/cm^2^ while demonstrating lower toxicity to mammals [109]. For surface disinfection, 10 min of exposure to 254 nm UV-C completely inactivated PRRSV on common farm materials (e.g., wood, plastic, concrete, and cloth) [110]. This technology effectively interrupts both aerosol and contact transmission of PRRSV, providing a practical solution for rapid disinfection in swine barn settings. Viral aerosols exhibit high sensitivity to UV radiation, and even under conditions of elevated humidity, their inactivation efficacy remains largely uncompromised—unlike what is observed with certain bacterial aerosols. Consequently, UV disinfection demonstrates significant practical utility in controlling the airborne transmission of viruses across diverse farm environments [111].

UV light has the advantages of being inexpensive, easy to use, highly effective in sterilization, and free of chemical residues [112,113,114]. However, it can cause damage to human skin and eyes. Additionally, UV light has weak penetration and a limited range of action. For large-scale air disinfection, a large number of UV lamps are required, which can be highly energy-consuming [114].

##### High-Voltage Electrostatic Adsorption Method

High-voltage electrostatic adsorption utilizes a high-voltage electrostatic device to generate static electricity, which causes microbial carrier particles in the air to settle and slide down. Subsequently, certain adsorbent materials are used to adsorb these microbial carrier particles to achieve the purpose of sterilization and disinfection. This purification method combines electrostatic purification with adsorption purification and is commonly used after the coarse filtration section of combined air conditioning units, capable of simultaneously removing gaseous and particulate pollutants [115]. The electrostatic field generated by the high-voltage electrostatic device, at potentials several thousand to tens of thousands of volts below, induces corona discharge from the discharge electrode metal wire. This process produces a large number of gas particles for aerosol particle charging, leading to the charging of dust and bacteria, migration of floating dust, and deposition on the electrode plate, thereby achieving dust and bacteria removal. Additionally, while generating a high-voltage electrostatic field, electrostatic adsorption typically produces a significant amount of ozone [116]. Higher concentrations of ozone can be harmful to humans, so this technology is often used in conjunction with ozone adsorption or decomposition devices. Moreover, the effective reduction of microbial numbers in the air by electrostatic adsorption mainly relies on the adsorption action on the negative electrode plate, making it difficult to directly inactivate microorganisms.

##### Ionizing Radiation

Ionizing radiation typically employs high-energy X-rays or gamma rays, which can disrupt DNA through direct energy deposition or secondary interactions with surrounding atoms and molecules. Particularly, secondary interactions with water molecules generate hydroxyl radicals, accounting for approximately 90% of DNA damage [117]. Both direct and indirect interactions can lead to significant double-strand breaks, thereby inactivating microorganisms.

Numerous studies have demonstrated the effectiveness of ionizing radiation in virus inactivation. Feldmann et al. used gamma rays generated from a cobalt-60 source to irradiate several viruses at different doses. The results showed that SARS-CoV could be completely inactivated at the lowest irradiation dose of 10 kGy [118]. Delrue et al. conducted research indicating that PRRSV can be inactivated by γ-irradiation with a minimum effective dose of 0.25 kGy, and a dose of 1.5 kGy is recommended to ensure complete inactivation of the virus [119,120]. Jebri et al. discussed the relationship between gamma-ray sensitivity and the infected medium in their study, revealing that humidity and radiation dose are negatively correlated when achieving the same sterilization effect [121]. Therefore, it may be necessary to adjust the radiation dose according to the specific application context.

Ionizing radiation has several advantages, including strong penetration ability, the ability to sterilize without opening packaging, and no residual or induced radioactivity after irradiation [122]. These principles hold true across its applications, including in livestock farming, where it is primarily used for feed sterilization to eliminate pathogens like *Salmonella* without compromising packaging integrity or leaving harmful residues [123]. However, during sterilization, gamma/X-rays may also damage the treated polymers, leading to embrittlement and loss of strength, which can affect their functionality. It is worth noting that while this poses a significant concern for medical devices, the impact on livestock farming is different; the primary concern shifts to the potential degradation of heat-sensitive nutrients (e.g., vitamins) in feed, rather than the embrittlement of plastics [124]. When applied to air disinfection, this technology also requires an unoccupied environment and strict control of the radiation dose—a universal safety requirement that is equally critical in livestock settings to ensure operational safety and achieve the desired microbiological efficacy without adversely affecting the target material [125].

##### Laser Irradiation

Laser technology has demonstrated significant potential in air purification and disinfection. By integrating laser-based disinfection devices into the ductwork of HVAC (heating, ventilation, and air conditioning) systems, real-time sterilization of circulating air can be achieved [126], effectively inactivating airborne bacteria, viruses, and other microbial contaminants.

The bactericidal mechanisms of laser technology primarily involve three distinct modes of action: (i) Photothermal effects: The concentrated delivery of laser energy induces rapid localized temperature elevation, causing hyperthermic damage to bacterial cell membranes. This thermal catalysis triggers cellular lysis and biomolecular decomposition, ultimately leading to microbial inactivation [127]. (ii) Photochemical interactions: Laser photons interact with cellular components, facilitating the cleavage of chemical bonds or the catalytic generation of free radicals. These reactive species disrupt critical biomolecules such as DNA and structural proteins, thereby inducing lethal oxidative stress [128]. (iii) Photomechanical shock: The high energy density of pulsed laser irradiation generates transient pressure waves, inducing mechanical stress that deforms or ruptures microbial cell walls and organelles, resulting in irreversible loss of viability [128].

However, the application of laser disinfection is not without challenges. High-energy laser systems often entail elevated capital and operational costs, while improper handling poses risks of ocular or dermal injury. Furthermore, microbial susceptibility to laser inactivation varies significantly across species, necessitating wavelength-, power-, and exposure time-specific optimization to target pathogens effectively. Consequently, the development and deployment of laser disinfection technologies require a multidisciplinary approach that balances technical feasibility, cost-effectiveness, and compliance with safety protocols to ensure efficient, safe, and sustainable implementation in air quality management systems [129,130].

##### Microwave Radiation

Microwaves, as a type of non-visible electromagnetic wave, are increasingly being utilized in the field of disinfection, demonstrating unique advantages of high efficiency, speed, and non-contact operation. In disinfection technologies, the commonly used microwave frequencies are (915 ± 25) MHz and (2450 ± 50) MHz [131]. These specific frequencies of microwaves can penetrate deeply into objects and interact strongly with polar molecules within the material, particularly water molecules, thereby achieving sterilization and disinfection effects. The mechanisms of microwave disinfection mainly involve three integrated effects: molecular heating, cellular structural disruption, and quantum effects [132].

The rapid penetration of microwaves and the direct generation of heat through molecular friction are fundamental principles of their sterilization and disinfection capabilities. When microwave radiation acts on microorganisms, its energy can quickly penetrate the microbial body and interact with internal polar molecules, such as water molecules. Under the influence of the microwave field, these polar molecules rotate at high speeds and rub against each other, generating heat and causing a rapid increase in the internal temperature of the microorganism. This sustained high-temperature environment disrupts the living conditions of microorganisms, destabilizes their cell membranes, affects enzyme activity and metabolic processes, and ultimately leads to microbial inactivation [133,134].

Simultaneously, the field effect of microwaves also plays a significant role in the sterilization process. When a biological entity is placed in a microwave field, microorganisms experience intense shocks and oscillations [135]. This physical action can disrupt the outer cellular structures, such as cell walls and membranes. The destruction of cellular structures increases cell permeability, disturbs the balance of materials inside and outside the cell, and may cause leakage of intracellular substances while allowing harmful external substances to invade, disrupting the normal balance of materials within the cell. This disruption of cellular structure and function ultimately leads to the disintegration and fusion of the cytoplasm, causing microbial death [132].

Additionally, the quantum effects in the microwave field are also part of the sterilization mechanism. Under the influence of the microwave field, polar molecules such as water molecules may become excited and generate reactive oxygen species with strong oxidizing properties, such as hydrogen peroxide and hydroxyl radicals. These radicals, with their high reactivity, can damage various biomacromolecules within the cell, such as proteins and nucleic acids, creating cytotoxic effects. This quantum effect not only directly destroys the cellular structure of microorganisms but also indirectly affects the normal progression of various biochemical processes within the cell, thereby achieving sterilization [132].

#### 5.2.2. Chemical Disinfection

Chemical disinfection technologies, including the use of chemical disinfectants, traditional Chinese herbal disinfectants, ozone, photocatalytic oxidation, and plasma, primarily rely on chemical reactions to disrupt the biological activity of microorganisms.

##### Chemical Sanitizer

Commonly used chemical disinfectants include chlorine-containing disinfectants, iodine-containing disinfectants, peroxides, ethylene oxides, alcohols, and other disinfectants [136]. The active ingredients in disinfectants interact with large molecules on the outer layer of microorganisms, such as proteins, glycoproteins, and membrane lipids, and inhibit the synthesis of RNA, DNA, and proteins, thereby killing the microorganisms [137]. It has been shown that the type of disinfectant, dosage, contact time, pH value, temperature, and solution composition are all key factors affecting the efficiency of virus inactivation in the air [138].

Research on disinfectant efficacy against pathogens highly relevant to livestock farms has yielded critical insights. For instance, a study focusing on ASFV—a virus of paramount concern in pig farming due to its extreme resilience—found that sodium hypochlorite at a concentration of 0.5% was required to completely inactivate the virus on surfaces within 10 min [139]. Similarly, against AIV, which poses a significant threat to poultry operations, peracetic acid at 0.2% and hydrogen peroxide at 1.0% have been demonstrated to effectively destroy viral infectivity within 5 min under farm-relevant conditions [140,141]. These findings underscore the necessity of selecting disinfectants and concentrations based on the specific, high-consequence pathogens present in the livestock environment.

Owing to their simplicity, cost-effectiveness, and potent sterilization capabilities, chemical disinfectants have garnered extensive application in the thorough disinfection of the livestock living environment. Disinfection is predominantly carried out via spraying or steaming. However, the use of chemical disinfectants inevitably leaves chemical residues in indoor spaces. Therefore, when choosing the type and concentration of the disinfectant, it is essential to fully consider the potential harm that the disinfectant may cause to the animals themselves [142,143].

##### Chinese Herbal Disinfectant

Traditional Chinese herbal disinfectants, due to their natural sourcing, safety, non-toxicity, and low likelihood of inducing resistance, can achieve safe and effective disinfection even in occupied spaces, posing no harm to humans or the environment [144]. In recent years, they have garnered increasing attention.

Research by Orimaye et al. indicates that extracts and bioactive components from traditional Chinese herbs significantly control common pathogenic bacteria such as *Salmonella* in broiler chicken farming, effectively reducing the number of bacteria in the gut and thereby lowering the risk of carcass contamination during slaughter [145]. Yu et al. demonstrated that herbal disinfectants are more environmentally friendly, less likely to produce harmful residues, and cause less irritation to the environment and animals, thus helping to reduce pollution in farming environments [64]. Additionally, it was found that in pig farms, herbal disinfectants can serve as a supplement or alternative to conventional disinfectants. Spraying or fumigating with herbal extracts can reduce bacterial and fungal aerosols in pig barns, thereby lowering the risk of disease transmission [64]. However, herbal disinfectants also have their drawbacks, such as complex extraction processes, high costs, and sometimes unstable effects, which have long constrained the development and promotion of herbal air disinfectants.

##### Ozone Disinfection

Ozone disinfection is a highly efficient, broad-spectrum, and eco-friendly sterilization method characterized by thorough microbial inactivation and uniform spatial coverage. It can disrupt and oxidize the cell walls, cell membranes, amino acids, proteins, nucleic acids, and other substances of microorganisms through both direct and indirect pathways. Additionally, it can interfere with the growth, metabolism, and reproductive processes of cells, ultimately rendering them inactive [146]. The efficacy of ozone disinfection is influenced by multiple parameters, including ozone concentration, exposure duration, RH, and microbial species [147]. Empirical studies demonstrate a positive correlation between disinfection efficiency and these variables within optimal ranges: higher ozone concentrations, prolonged exposure durations, and elevated RH levels synergistically enhance microbial lethality. Notably, the humidity-dependent generation of hydroxyl radicals (•OH) under elevated RH conditions amplifies oxidative stress, further improving inactivation kinetics against resistant pathogens [148,149].

Ozone is a strong oxidizing agent commonly used in chemical laboratories, known for its broad-spectrum and rapid inactivation of microorganisms [150]. Ozone disinfection refers to the process of purifying and disinfecting air using ozone generated by an ozone generator. This purification method can effectively alleviate problems such as microbial and algal growth, corrosion, and scaling in direct evaporative cooling air-conditioning recirculating cooling water systems [151]. The ozone disinfection and sterilization process is a biochemical oxidation reaction. The purification principles are threefold: (i) Oxidative decomposition of the enzymes required for microbial synthesis of glucose, leading to microbial inactivation and death. (ii) Destruction of microbial organelle DNA or RNA, disrupting their metabolic systems and causing microbial death. (iii) Penetration of the cell membrane structure, acting on the lipoproteins of the outer membrane and the lipopolysaccharides inside, causing bacteria to undergo permeability distortion and dissolve to death.

In the livestock farming context, where controlling pathogenic bacteria, viruses, and fungi is critical for animal health and biosecurity, ozone has demonstrated excellent inactivation efficacy [152,153]. Zhang et al. experimentally evaluated the efficacy of ozonized water in inactivating ASFV. The results indicated that the titer of ASFV on contaminated surfaces decreased by 3 orders of magnitude after treatment with ozonized water concentrations of 10 mg/L for 1 min, and the same virus titer was completely inactivated by 20 mg/L ozonized water within 3 min [154]. This evidence supports the use of ozone as a highly effective disinfectant for surface decontamination in livestock facilities, helping to mitigate disease transmission.

Ozone has broad-spectrum bactericidal effects and is highly effective in sterilization. The production of ozone is simple, cost-effective, and rapid. Importantly, it can effectively diffuse to any part of a room, including areas that may be difficult to reach using traditional liquid and manual cleaning methods [155]. However, its drawbacks include corrosiveness to certain material surfaces and potential toxicity to humans with prolonged exposure, limiting its use to unoccupied environments for disinfection purposes. Therefore, ozone is rarely used as a sole disinfectant. Instead, it is commonly combined with other disinfection methods to create a synergistic effect, thereby achieving better disinfection results [146,156].

##### Photocatalytic Oxidation Method

Photocatalytic disinfection technology refers to the generation of highly oxidative active components, such as hydroxyl radicals, by photoactive oxides under UV irradiation. These active components disrupt the structure of biological cells, thereby achieving the inactivation of microorganisms [157,158]. Photocatalytic disinfection is characterized by its high efficiency, low cost, and safety. It can be used to inactivate a variety of microorganisms, including bacteria, viruses, and molds, with varying degrees of effectiveness [159,160].

There are three common mechanisms of photocatalytic oxidation for degradation: (i) Oxidation of respiratory enzymes to inhibit the respiration of living organisms. (ii) Oxidation and destruction of cell walls and cytoplasmic membranes, leading to the loss of semipermeability and inactivation of the organism. (iii) Destruction of nucleic acids in living organisms, inhibiting the replication of genetic material and metabolic functions, resulting in complete inactivation [161]. Chemical materials suitable for photocatalytic oxidation purification include titanium dioxide (TiO_2_), zinc oxide, cadmium sulfide, and tungsten trioxide.

Nano-TiO_2_, with its high catalytic activity, strong oxidizing power, stable chemical properties, low cost, and strong corrosion resistance, has become the most widely used photocatalytic material to date. It is suitable for air and surface disinfection [162]. In practical applications, attention should be paid to the impact of dust and other factors on the performance of the catalyst. The decline in catalyst performance over long-term use can lead to reduced disinfection effectiveness, so it is necessary to monitor the catalyst’s performance in a timely manner.

##### Plasma Disinfection

Plasma, a distinct state of matter beyond the conventional solid, liquid, and gas phases, refers to a highly ionized gaseous cloud generated through gas ionization under thermal or electromagnetic excitation. It comprises a dynamic mixture of electrons, ions, neutral atoms/molecules, reactive free radicals, and electromagnetic radiation [163]. Termed “plasma” due to the numerical equivalence of total positive and negative charges, its generation methods include electrical discharge, radiation exposure, ionization, and laser excitation. Plasma disinfection, characterized by rapid and high-efficiency microbial inactivation, is applicable to both surface and airborne pathogen control. Its biocidal mechanism involves synergistic physicochemical interactions: reactive oxygen species (ROS) and atomic oxygen within the plasma induce oxidative damage to microbial cell membranes, proteins, and DNA, while high-energy particles physically disrupt cellular integrity, leading to irreversible inactivation [164].

Plasma disinfection is highly effective against common livestock pathogens. Its efficacy in sanitizing air within barns and enclosed livestock spaces increases with higher temperature, humidity, and exposure duration, within optimal ranges [165]. Critically for disease control, Clack et al. reported superior inactivation of porcine reproductive and PRRSV—a swine-specific pathogen with no zoonotic potential—in airborne environments via plasma-generated reactive species compared to conventional ozone disinfection, achieving over 95% viral inactivation within seconds [166]. This finding highlights the potential of plasma technology to mitigate airborne viral transmission in challenging agricultural settings such as livestock farms, offering a powerful tool for enhancing biosecurity.

Unlike conventional chemical-based methods, plasma disinfection exhibits multifunctional adaptability, with sterilization modes varying by plasma generation technique (e.g., dielectric barrier discharge *vs*. atmospheric-pressure plasma jets). This versatility enables its classification as a comprehensive and universally applicable air purification strategy. Critically, plasma technology supports continuous disinfection in occupied spaces, demonstrating real-time bacterial load reduction even during surgical procedures, thereby addressing infection control challenges in clinical settings [167].

#### 5.2.3. Physical Barriers

Microbial aerosols serve as critical vectors for pathogen transmission in livestock farming environments, with their particle size range (0.1–100 μm) enabling long-distance migration via aerodynamic forces. Physical barriers employ a tripartite mechanism—spatial segregation, mechanical interception, and directional airflow control—to disrupt transmission chains. Spatial isolation barriers (e.g., metal fences, mesh partitions) directly separate highly contaminated zones (manure treatment areas) from clean areas (housing sections), achieving > 50% interception efficiency for particles ≥ 5 μm (e.g., fungal spores, feed dust) [168]. Such barriers require species-specific designs: poultry houses demand high-density mesh (aperture ≤ 2 cm) to block feather-derived aerosols, whereas swine facilities necessitate reinforced floor barriers to prevent excreta splashing [169]. Negative-pressure ventilation systems maintain stable indoor pressure (≥15 Pa), forcing pathogen-laden aerosols through HEPA filters to block 99% bacterial aerosol leakage [170]. Experimental data demonstrate that HEPA installation in tiered poultry houses reduces airborne aerobic bacteria to 1.5 × 10^3^ CFU/m^3^—an 80% decrease compared to untreated groups [50].

Advanced technologies focus on precision interception and source control. The Individual Cage Ventilation (ICV) system provides dedicated air ducts for each cage, eliminating cross-contamination and resulting in a 62% reduction in H5N1 antibody positivity rates in duck houses, alongside a 28% increase in T-lymphocyte transformation rates [55]. Bio-sealed composting technology employs plastic membranes for physical containment of carcasses, coupled with high-temperature (>55 °C) pathogen inactivation, achieving complete elimination of Salmonella and other pathogens within 147 days [171]. Notably, physical barrier efficacy is environmentally modulated: fungal aerosol concentrations increase 5.8-fold during autumn/winter [36], necessitating augmented activated carbon filtration layers to intercept elevated mold spores, with concurrent mesh density adjustments to accommodate humidity variations [19].

### 5.3. Balance Between Insulation and Ventilation

In livestock production systems, achieving equilibrium between thermal insulation and ventilation is pivotal to ensuring animal welfare and optimizing operational efficiency. Producers frequently encounter a paradoxical dilemma: maintaining housing warmth by minimizing ventilation frequency to mitigate cold stress on livestock, while excessive insulation inadvertently compromises air quality through elevated microbial aerosol concentrations, thereby jeopardizing animal health and productive performance. Consequently, identifying the optimal synergy between thermal regulation and air exchange constitutes a critical challenge in modern husbandry management [172].

Primary to formulating balanced strategies is comprehending the physiological impacts of these parameters. Thermal insulation proves indispensable for maintaining homeothermy, reducing metabolic energy expenditure, and supporting growth trajectories, particularly under winter conditions or in cold climates [173]. However, hyperinsulation-induced air stagnation elevates concentrations of airborne pathogens, noxious gases (e.g., NH_3_, CO_2_), and particulate matter, exacerbating risks of respiratory pathologies and immunosuppression. Conversely, judicious ventilation facilitates pollutant dilution and microbial load reduction, though excessive air exchange may precipitate thermal shock through rapid temperature fluctuations, impairing feed conversion efficiency and production metrics [174,175]. Strategic adaptations should therefore be implemented through seasonal and climatic lenses: during cold phases, prioritized insulation must integrate controlled ventilation protocols (e.g., intermittent mechanical ventilation) to concurrently maintain thermoneutral zones and evacuate toxic volatiles. In warmer periods, augmented ventilation rates via natural convection or forced-air systems serve dual purposes of thermal mitigation and microbial aerosol abatement [64].

The integration of intelligent environmental control systems represents a paradigm-shifting approach to this equilibrium. Contemporary facilities employ networked sensors monitoring real-time temperature, relative humidity, and airborne particulate counts, interfaced with automated ventilation actuators and HVAC systems [176]. This cyber-physical architecture enables precision environmental modulation through algorithmic regulation of vent apertures and thermal conditioning, achieving dual objectives of climate resilience and energy optimization. Concurrently, architectural innovations enhance this balance through double-walled insulation composites and computational fluid dynamics-optimized vent configurations, ensuring laminar airflow patterns that prevent direct draught exposure while maximizing air exchange efficiency. Spatial planning interventions, including reduced stocking densities and zonal segregation, further ameliorate microenvironmental conditions and suppress aerosol generation [177,178].

Systematic environmental auditing remains imperative for sustained optimization. Regular monitoring of critical parameters (ambient temperature gradients, humidity profiles, NH_3_ ppm) informs dynamic adjustments to ventilation-insulation protocols. Furthermore, zootechnical considerations (including growth phase-specific requirements and health status monitoring) necessitate adaptive modifications to stocking densities and air exchange rates, ensuring perpetual maintenance of homeostasis within the animals’ physiological tolerance thresholds [179,180]. This multidimensional approach, synthesizing engineering solutions with biological monitoring, establishes a robust framework for sustainable intensive production systems.

## 6. Closing Remarks

In the context of intensification and scaled-up production systems within modern animal husbandry, the control of microbial aerosols has transcended its traditional role as a disease management tool, evolving into a pivotal strategic initiative to enhance livestock productivity and ensure the sustainable development of the industry. Confronted with this invisible yet multifaceted challenge, the livestock sector must adopt a proactive stance by constructing a comprehensive mitigation framework that integrates cutting-edge technologies, eco-friendly mitigation philosophies, and precision management practices. Scientific husbandry protocols (including optimized rearing environments, precision nutrition formulations, and data-driven holistic health management) can synergistically improve animal welfare, accelerate growth performance, and reduce disease risks associated with microbial aerosol exposure. For instance, enhanced ventilation systems, balanced dietary interventions, and routine health surveillance coupled with preemptive disease prophylaxis not only elevate production efficiency but also drive the industry’s transition toward green, low-carbon operational models.

In conclusion, microbial aerosol mitigation is not merely a reactive measure against pathogen transmission but a cornerstone of the livestock sector’s green transformation and sustainable evolution. By systematically implementing standardized aerosol sampling and detection technologies, eco-friendly mitigation strategies, and evidence-based management protocols, the industry can safeguard animal health, optimize production metrics, and uphold ethical husbandry standards. This integrated approach lays a robust foundation for achieving health-centric, high-efficiency, and environmentally responsible livestock production. Through such advancements, the sector is poised to transition from conventional paradigms to intelligent, sustainability-oriented systems, propelling the industry toward a healthier, more efficient, and environmentally sustainable future.

## Data Availability

No new data were created or analyzed in this study.
